# Chromatin Signature Identifies Monoallelic Gene Expression Across Mammalian Cell Types

**DOI:** 10.1534/g3.115.018853

**Published:** 2015-06-18

**Authors:** Anwesha Nag, Sébastien Vigneau, Virginia Savova, Lillian M. Zwemer, Alexander A. Gimelbrant

**Affiliations:** Department of Cancer Biology and Center for Cancer Systems Biology, Dana-Farber Cancer Institute; and Department of Genetics, Harvard Medical School, Boston, Massachusetts 02215

**Keywords:** gene expression, epigenetics, chromatin

## Abstract

Monoallelic expression of autosomal genes (MAE) is a widespread epigenetic phenomenon which is poorly understood, due in part to current limitations of genome-wide approaches for assessing it. Recently, we reported that a specific histone modification signature is strongly associated with MAE and demonstrated that it can serve as a proxy of MAE in human lymphoblastoid cells. Here, we use murine cells to establish that this chromatin signature is conserved between mouse and human and is associated with MAE in multiple cell types. Our analyses reveal extensive conservation in the identity of MAE genes between the two species. By analyzing MAE chromatin signature in a large number of cell and tissue types, we show that it remains consistent during terminal cell differentiation and is predominant among cell-type specific genes, suggesting a link between MAE and specification of cell identity.

A significant fraction of mammalian genes are under control of epigenetic mechanisms that cause their transcription to occur exclusively or predominantly from one allele. In addition to approximately 1000 X-linked genes that are subject to chromosome-wide X inactivation in female cells (Morey and Avner 2011) and approximately 100 known imprinted genes (Morison *et al.* 2005; Kelsey and Bartolomei 2012), up to 15% of human and mouse autosomal genes are subject to mosaic monoallelic expression (MAE) (Savova *et al.* 2013; Eckersley-Maslin and Spector 2014). Genes subject to MAE can be expressed from the maternal allele in one cell and from the paternal allele or from both alleles in a neighboring cell in the same individual (Gimelbrant *et al.* 2007). This allelic expression is mitotically stable in clonal cell lines and independent between loci, enabling vast epigenetic heterogeneity within cell populations. Furthermore, when the two alleles encode different products, MAE can profoundly affect cell function. A better understanding of the role and mechanisms of MAE should thus lead to new insights into the relationship between genotype and phenotype.

We previously showed that genes with gene−body enrichment in both H3K27me3 and H3K36me3 in human lymphoid cells were highly likely to be MAE (Nag *et al.* 2013). Because this chromatin signature does not require polymorphisms or monoclonal cell culture, it bypasses limitations of other approaches relying on direct measurement of allelic expression, and allows the investigation of genome-wide MAE patterns *in vivo*.

Here, we use the chromatin signature approach for comparative analysis of MAE in cells and tissues of distinct embryonic origins. We show that the MAE chromatin signature is conserved in mouse, orthologous genes tend to share propensity for MAE in mouse and human, and the same functions are associated with MAE genes in both species. Comparison of MAE profiles in different mouse cell lineages also suggests that MAE is established in a lineage-specific manner and stably maintained throughout terminal cell differentiation.

## Materials and Methods

### Cell culture

Monoclonal cell lines used in this work have been previously described in (Zwemer *et al.* 2012) and are listed in Supporting Information, Table S1. For simplification we have renamed the lines as S1Cs-A5, Abl.1; S1Cs-A7, Abl.2; S1Cs-F1, Fib.1; S1Cs-F2, Fib.2. Cells were grown at 37° in presence of 5% CO_2_. Abl.1 and Abl.2 lines were cultured in Roswell Park Memorial Institute medium (*i.e.*, RPMI) complemented with 20% fetal bovine serum, 1× penicillin-streptomycin-glutamine (10378-016; Gibco) and 1× GlutaMAX Supplement (35050-061; Gibco). Fib.1 and Fib.2 lines were cultured in Dulbecco’s Modified Eagle Medium complemented with 10% fetal bovine serum, 1× penicillin-streptomycin-glutamine and GlutaMAX Supplement.

### RNA-Seq

PolyA purified RNA was reverse transcribed using random primers to prepare strand-specific Illumina compatible libraries, following methods described in Parkhomchuk *et al.* (2009) and Nag *et al.* (2013). Libraries were sequenced using Illumina Hi-Seq platform (SE50).

A single-nucleotide polymorphism (SNP)-masked reference for 129CASTF1 transcriptome was generated from mm9 mouse genome assembly, using an in-house pipeline implemented in Awk, by removing nontranscribed regions based on GTF annotation and masking SNP loci imputed from parental strain genome. The libraries from Abl.1 and Abl.2 lines yielded 43 million and 28.4 million reads, respectively, whereas the libraries from Fib.1 and Fib.2 lines had 41 million and 61 million reads. All reads were mapped against the SNP-masked reference using Bowtie 2, with default parameters. To counteract disparities in duplication rate and potential allele-specific artifacts, only unique reads were used. Mapped read counts for the maternal and paternal allele of each SNP were obtained using Samtools (Li *et al.* 2009) and custom Perl scripts. Allelic bias was statistically identified from the resulting SNP allelic counts with in-house Matlab analysis pipeline (Nag *et al.* 2013). Briefly, false-discovery rate corrected binomial p-value lower than 0.05, together with 2:1 bias, were considered evidence for monoallelic expression, whereas a positive equivalence test was evidence for biallelic expression. Results from allelic expression bias analysis are presented in Table S3.

### ChIP-Seq

Chromatin immunoprecipitation sequencing (ChIP-Seq) on clonal cell line Abl.1 was performed as described previously (Bernstein *et al.* 2006; Mikkelsen *et al.* 2007; Nag *et al.* 2013). In summary, cells were fixed with 1% formaldehyde for 5 min at 37°. Fragmentation was performed using Covaris sonicator. An aliquot of sheared chromatin was saved as input control. Immunoprecipitation was performed with Anti-H3K27me3 antibody (ABE44; Millipore, Billerica, MA) and anti-H3K36me3 antibody (AB9050; Abcam, Cambridge, MA and UK), using Protein-A Sepharose beads. ChIP-Seq libraries were prepared using NEBNext ChIP-Seq library prep reagent set for Illumina (NEB E6200S) following manufacturer’s instructions. Barcoded libraries were sequenced using Illumina HiSeq platform (SE50).

The H3K27me3, H3K36me3 and input control reads were mapped to the mm9 genome using Bowtie 2 with default parameters (Langmead and Salzberg 2012). Library sizes were of 32, 46, and 57 million unique reads, respectively, with alignment rate of 92% in each case. Duplicate reads were removed in accordance with standard practice for ChIP-Seq data (Carroll *et al.* 2014).

### MaGIC

To calculate gene-body signal for H3K27me3 and H3K37me3, files listed in Table S1 were first converted to wig format by the use of a combination of custom Perl script, the bigWigToBedGraph utility from UCSC (http://hgdownload.cse.ucsc.edu/admin/exe/linux.x86_64/) (Kent *et al.* 2010), Samtools (Li *et al.* 2009), and the rsem-bam2wig program (Li and Dewey 2011). Gene-body signal was calculated, for each mark and the corresponding input, by summing signal over the length of the longest transcript for each autosomal gene. Signals for H3K27me3 and H3K36me3 were normalized to input signal, or to the transcript length if input was not available. Signal from various replicates was averaged when necessary. After discarding transcripts with no input signal, quantile rank was calculated for each normalized signal, using average rank in case of tie.

The alternating decision tree classifier was trained on the human GM12878 dataset using the same training set for allelic expression and using Weka 3.7.3 (Hall *et al.* 2009) with the same settings (neutral classifier) as described previously (Nag *et al.* 2013). The only notable differences were that we used an updated version of the GM12878 dataset that includes three instead of two replicates (Table S1) and, more importantly, that we trained the classifier on the quantile rank instead of the base 2 logarithm of normalized gene-body signal. As expected with an alternating decision tree classifier, genes predicted as MAE in GM12878 showed only minor changes compared to our previous study, consistent with the use of slightly different ChIP-Seq datasets. To note, in this context, the use of quantile rank is equivalent to quantile normalization, but without requiring a reference dataset. Consequently, the newly trained classifier can be readily applied to other datasets with gene−body signal expressed as quantile-rank, even when absolute values are on different scales due to experimental or data processing differences, as illustrated on Figure S1.

As part of the Monoallelic Gene Inference from Chromatin (MaGIC) procedure, after applying the classifier to a new dataset, we filtered out genes shorter than 2.5 kb, for which chromatin signature was not reliable, and low expressed genes, using median expression as the threshold (Figure S2). For gene length, we used the length of the longest transcript in mm9 or mm8 genome assembly, depending on which assembly had been used for the alignment (Table S2). To calculate relative transcript abundance and estimate the median expression, we integrated transcript signal over the length of the longest transcript for each gene and normalized to transcript length by using the same pipeline as for H3K27me3 and H3K36me3 gene-body signal. Alternatively, for clone Abl.1, we used FPKM values calculated using Rsem software as a measure of transcript abundance (Li and Dewey 2011). Expression rank was used instead of RPKM values because the biological meaning of the RPKM measure can vary between cell types (Mortazavi *et al.* 2008). To put it in the context of our previous analysis, in the human GM12878 cells, median expression corresponded to RPKM≈1 (Nag *et al.* 2013).

For subsequent analysis, we only kept datasets that passed a simple test to ensure minimal dynamic range of ChIP-Seq enrichment (Figure S3). Using median H3K27me3 and H3K36me3 enrichment as the threshold, we divided genes in four groups on the basis of the relative enrichment of these two marks (low H3K27me3 and high H3K36me3, high H3K27me3 and low H3K36me3, low H3K27me3 and low H3K36me3, and high H3K27me3 and high H3K36me3). We then asked that the number of genes in the first two groups is at least 50% greater than the number of genes in the two remaining groups. Datasets matching this criteria show a distribution of genes that appears visually similar to that in the human lymphoblastoid cells used to train the classifier.

### Comparison of monoallelically expressed genes between mouse and human

MAE genes were identified from mouse CD43^−^ B cells and human peripheral blood mononuclear cells datasets using the MaGIC pipeline (Table S1, Table S2, and Table S4). Orthologous mouse and human genes were determined using the HomoloGene database (Sayers *et al.* 2011). In this database, some genes in one species are mapped to multiple genes in the other species. For consistency and simplicity, only genes that had a single ortholog in each species were considered. Hypergeometric distribution was calculated with the online tool http://www.geneprof.org/GeneProf/tools/hypergeometric.jsp and used to assess the conservation of MAE as detailed in the main text. The list of conserved MAE genes uncovered by this analysis is shown in Table S5.

### GO analysis

Gene Ontology (GO) analysis was performed using GeneTrail (http://genetrail.bioinf.uni-sb.de/index.php) (Backes *et al.* 2007). Over-/underrepresentation analysis of GO categories was performed using only manually curated GO annotations. For a category to qualify as being significant, a minimum of 3 genes from that category had to be in the test dataset. The test dataset included genes predicted to be MAE and the background dataset included both genes predicted to be MAE and BAE. For each of these analyses, false-discovery rate (Benjamini and Hochberg) adjustment was performed (Benjamini and Hochberg 1995). Full analysis is reported in Table S6.

### Comparison of monoallelic expression profiles and clustering

To compare MAE profiles across tissues, Gower distance was computed using the Vegan package in R (Dixon 2003), based on the subset of genes that were expressed in both samples. Hierarchical clustering was performed using the “hstat” function from the R statistics package (RCoreTeam 2014), with the “average” (UPGMA) method.

### Correction for classifier accuracy

To correct for imperfect accuracy when inferring the number of MAE genes in a given number of samples (Figure S5B), we first computed for each gene, using simple combinatorial probability, the probability to be MAE in any number of samples given the number of samples where it was predicted to be MAE and the MAE prediction accuracy. We used a MAE prediction accuracy of 0.73, as calculated for the Abl.1 clone. We then summed, for each possible number of samples, the probability to be MAE of all genes. Each sum gives an estimate of the number of genes that are MAE in a particular number of samples; if it was smaller than 1, we considered that no gene was MAE.

## Results

### Chromatin signature in mouse primary cells identifies genes subject to MAE

To assess whether the H3K27me3/H3K36me3 gene-body chromatin signature is correlated with MAE in mouse, we compared results from a ChIP-Seq analysis (the “chromatin signature”) and a traditional analysis of allelic expression bias in clonal cell lines. We performed ChIP-Seq and RNA-Seq in a mouse B-lymphoid clonal cell line derived from 129S1/SvImJ × CAST/EiJ F1 mice, and immortalized with Abelson murine leukemia virus (clone Abl.1). The high density of SNPs between the two parental genomes allows for high-resolution allele-specific expression analysis (see the section *Materials and Methods*). Importantly, because of Abl.1’s monoclonal composition, this analysis provides an upper-bound estimate of how accurate is the correspondence between MAE called by chromatin signature and by allelic expression bias.

The classifier, trained on human datasets from our previous study, was applied to ChIP-Seq data as described (Nag *et al.* 2013), with several improvements to data processing and filtering (see the section *Materials and Methods*). We refer to the whole analytic pipeline consisting of the human-trained classifier and subsequent filters as the aforementioned Monoallelic Gene Inference from Chromatin (MaGIC).

**Figure 1:**
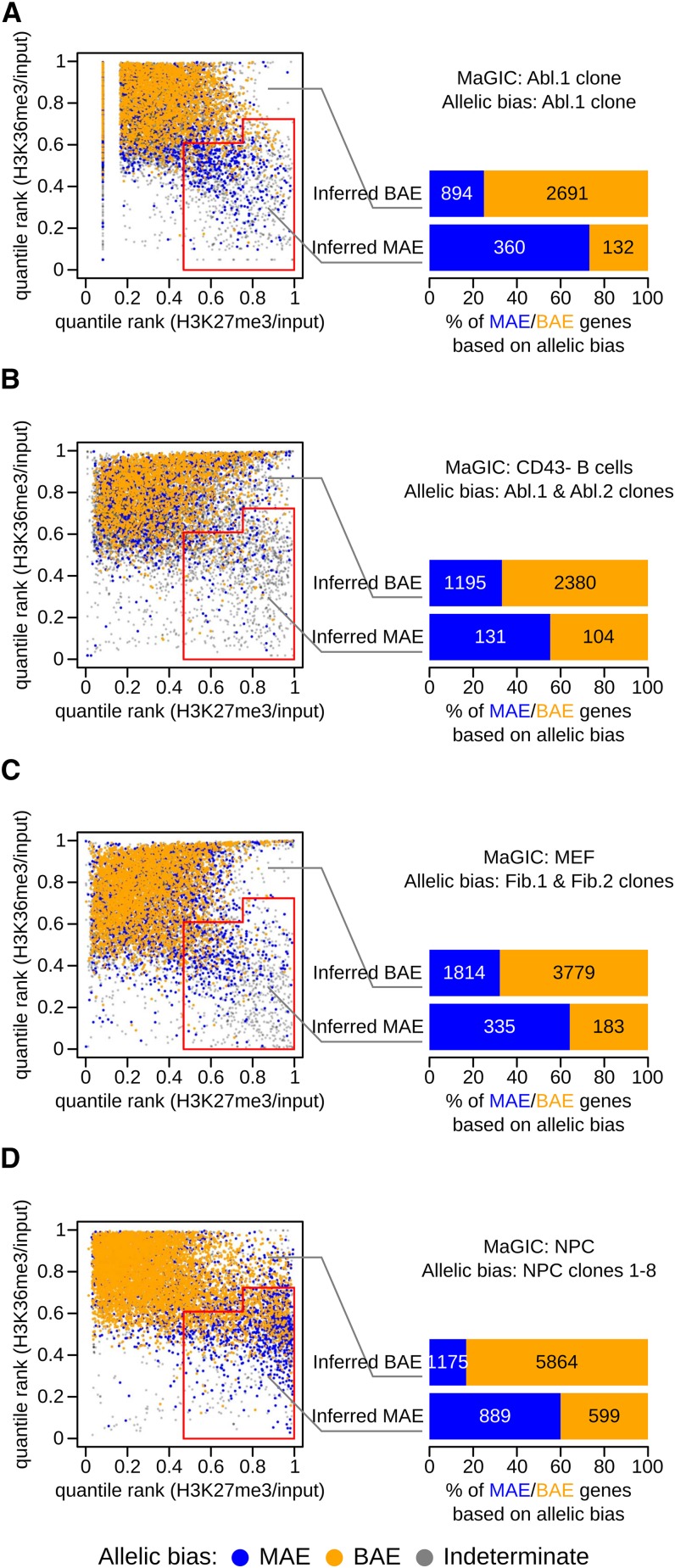
Chromatin signature is an informative proxy of mosaic monoallelic expression (MAE) in mouse cells from different lineages. (A) MAE state was inferred for genes with a particular combination of gene body signal for H3K27me3 and H3K36me3 ChIP-Seq, by applying the MaGIC pipeline in Abelson lymphoblast clone 1 from 129Sv/ImJ × CAST/EiJ F1 mouse (GSE67384; Table S1 and Table S2). Red line demarcates the area the classifier recognizes as being enriched with MAE genes (see *Materials and Methods*). Genes (represented as dots) are colored according to allelic expression analysis in the same clonal cell line: monoallelic (blue) or biallelic (gold) (Table S3). Genes with indeterminate allelic bias are shown as *gray* dots. The accuracy of the inference is summarized in the right panel, where genes are called monoallelic (blue) or biallelic (gold) on the basis of allele-specific analysis of RNA-Seq. (B−D) Same analysis as in (A), but with different sources of ChIP-Seq and RNA-Seq data, as indicated. (B) ChIP-Seq and RNA abundance data used in MaGIC pipeline were from CD43^−^ B cells (GSE31039; Table S1 and Table S2), and allele-specific RNA-Seq analysis was performed on two independent clonal cell lines from 129Sv/ImJ × CAST/EiJ F1 mice: Abl.1 and Abl.2 (GSE67384; Table S1 and Table S3; see main text for details). (C) ChIP-Seq and RNA abundance data from MEF cells (GSE12241; Table S1 and Table S2); allele-specific RNA-Seq analysis on two fibroblast clonal lines from 129Sv/ImJ × CAST/EiJ F1 mice: Fib.1 and Fib.2 (GSE67384; Table S1 and Table S3; see main text). (D) ChIP-Seq and RNA abundance data from neuronal progenitor cells (GSE33252; Table S1 and Table S2); allele-specific RNA-Seq analysis on eight clonal neuronal progenitor cell lines from 129Sv/ImJ × CAST/EiJ F1 mice (GSE54016; Table S1 and Table S3; see main text).

In Abl.1 clone, 4077 genes were assessable as either MAE or BAE by the use of both chromatin signature and allelic expression data. Of 492 genes classified as MAE with MaGIC, 73% were confirmed as true positive by RNA-Seq analysis, whereas of the remaining 3585 genes classified as BAE, 75% were confirmed as true negative [Figure 1A, Table S2, and Table S3; Fisher exact *P* < 2.2E-16; odds ratio (OR) = 8.2]. We conclude that the H3K27me3/H3K36me3 gene body chromatin signature is an informative proxy of monoallelic expression in mouse clonal B lymphoid cells and the MaGIC pipeline is up to 73% accurate for inferring MAE.

We next set out to evaluate whether MaGIC is accurate for inferring MAE in mouse non-clonal cells. RNA-Seq, however, cannot be used to directly measure MAE in nonclonal cell populations, because opposite allelic biases in different cells cancel out. We thus used RNA-Seq measurement of MAE in multiple clonal lines as the benchmark to approximate MAE in nonclonal cells of the same type: a gene was considered MAE if it was called MAE in at least one clone and BAE if it was called BAE in at least one clone and was not called MAE in any one clone. Note that due to the limited number of clones assessed and biological differences originating in the derivation process or the F1 genetic background (Table S1), the comparison with MaGIC provides a lower-bound estimate of the method’s potential accuracy in complex cell populations.

Using this approach, we assessed the accuracy of the MaGIC analysis in freshly isolated CD43^‒^ B cells, primary mouse embryonic fibroblasts, and *in vitro* differentiated neuronal progenitors. In B cells (of pure C57BL/6 background), MAE was inferred using ENCODE ChIP-Seq and RNA abundance data (Table S2), then compared to allele-specific analysis of RNA-Seq from the clone Abl.1 and a similar clonal line Abl.2 (from an independent 129S1/SvImJ × CAST/EiJ F1 cross; Table S3). Of 235 genes classified as MAE with MaGIC, 56% were confirmed by RNA-Seq, whereas of 3575 genes classified as BAE, 67% were confirmed, indicating that the MaGIC pipeline is also informative in freshly isolated cells of the B-lymphoid lineage (Figure 1B, Table S2, and Table S3; Fisher exact *P* < 1.5E-11; OR = 2.5).

Similarly, in mouse embryonic fibroblasts (129Sv-C57BL/6 background), MAE was inferred using ChIP-Seq (Mikkelsen *et al.* 2007) and ENCODE RNA abundance data, then compared with allele-specific analysis of RNA-Seq in two independent clones from 129S1/Sv × CAST/EiJ F1 SV40-immortalized fibroblasts, Fib.1 and Fib.2 (Table S1). Of 518 genes classified as MAE with MaGIC, 65% were confirmed by RNA-Seq, whereas of 5593 genes classified as BAE, 68% were confirmed, indicating that MaGIC is informative in primary fibroblasts (Figure 1C, Table S2, and Table S3; Fisher exact *P*-value < 2.2E-16; OR = 3.9).

Finally, in neuronal progenitors (129Sv-C57BL/6 background), MAE status was inferred using available ChIP-Seq and RNA abundance datasets (Tippmann *et al.* 2012), then compared with allele-specific analysis of RNA-Seq in eight neuronal progenitor clones from 129/Sv × CAST/EiJ F1 genetic background [(Gendrel *et al.* 2014); Table S1]. MaGIC classification as MAE was consistent with RNA-Seq for 60% of 1488 genes, whereas the classification as BAE was consistent for 83% of 7039 genes, indicating that MaGIC is informative in nonclonal neuronal progenitor cells differentiated *in vitro* (Figure 1D, Table S2, and Table S3; Fisher exact *P*-value < 2.2E-16; OR = 7.4).

Overall, these results show that the H3K27me3/H3K36me3 gene−body chromatin signature is an informative proxy of MAE in nonclonal cell populations from the lymphoid, mesenchymal, and neuroectodermal lineages. Conservatively, the MaGIC pipeline can be used to infer MAE in these lineages with 56–74% accuracy and BAE with 67–75% accuracy (OR between 2.5 and 8.2). The lower estimated accuracy in nonclonal cells may be partially due to the limited number of immortalized clones used for allelic expression analysis, especially compared with clonal complexity of primary cells. This factor likely accounts for the greater enrichment of confirmed MAE genes in MaGIC analysis of neuronal progenitors (8 clones; OR = 7.4) compared with lymphoblasts (two clones; OR = 2.5) and fibroblasts (two clones; OR = 3.9). Another source of both false-positive and false-negative calls could be potential influence of genetic background on the propensity of genes to be subject to MAE. Accordingly, when ChIP-Seq and RNA-Seq data were obtained on the same background (Figure 1A), the correspondence between chromatin signature and allelic bias was closer than otherwise (Figure 1, B−D). Discrepancies between MaGIC and RNA-Seq calls may also result from the confounding effect of particular expression patterns, as detailed in the *Discussion* section.

Also, note that CD43^‒^ B cells had pronounced chromatin signature of MAE despite being of pure genetic background, which indicates that the epigenetic states used by MaGIC analysis are independent of genetic differences between the two alleles. This finding is consistent with the detection of MAE using RNA-fluorescence in situ hybridization in pure genetic background, reported by Gendrel *et al.* (2014).

More generally, our analysis suggests that the chromatin signature is a molecular feature consistently associated with MAE in a wide variety of cell types across mammals.

### Chromatin signature of MAE is associated with orthologous genes and related functions in mouse and human

With both mouse and human genes exhibiting monoallelic expression, we examined the extent to which genes that show evidence of MAE in one species also display MAE in the other. Because MAE genes tend to have cell type-specific expression (Nag *et al.* 2013), such comparisons should be made on the basis of closely related cell types. We previously compared MAE between mouse and human immortalized lymphoid cells using low-resolution maps; 29 orthologous gene pairs could be assessed, nevertheless showing significant MAE conservation (Zwemer *et al.* 2012). The MAE chromatin signature allows us to carry out this comparison for a larger number of genes and in freshly isolated cells, avoiding possible artifacts due to cell culture.

**Figure 2:**
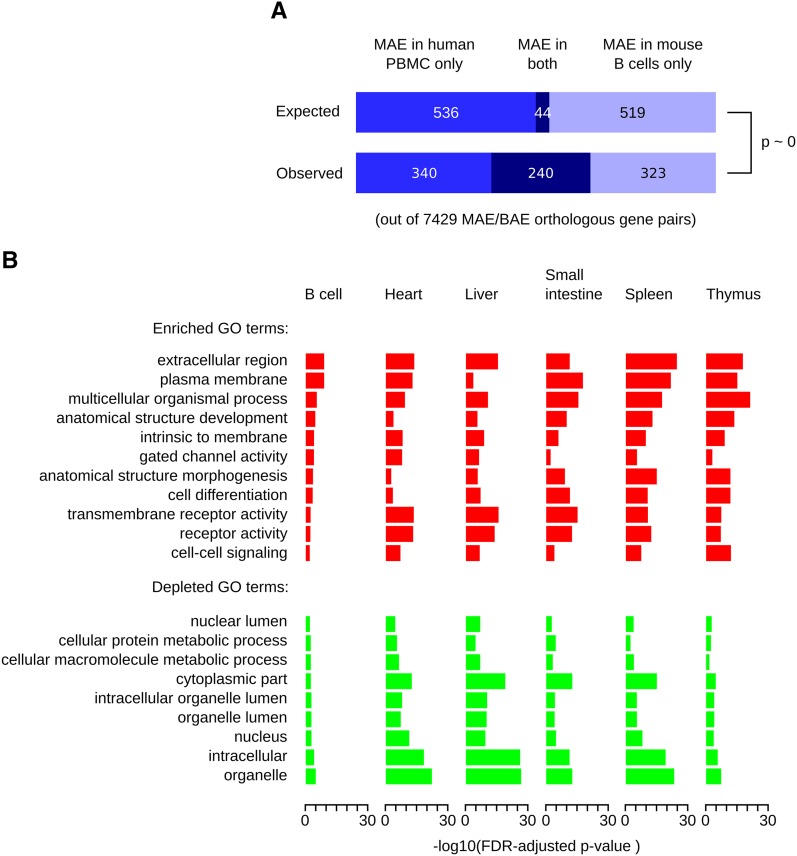
Common features of genes with mosaic monoallelic expression (MAE) chromatin signature in mouse and human. (A) Comparison of MAE in human and mouse genomes. The MAE state of orthologous genes was inferred in primary cells of the B lymphoid lineage using MaGIC pipeline. The number of genes that are MAE in only one or in both species is shown (bottom), as well as the expected distribution if the propensity of orthologous genes to be MAE were independent between species (top, see main text for details). The distributions are significantly different (hypergeometric p ∼ 0), indicating that the propensity of genes to be MAE is conserved. (B) Gene Ontology (GO) categories over-represented (red) and under-represented (green) among mouse genes with MAE chromatin signature. The categories that were over- and under-represented in human B-cells (Nag *et al.* 2013) are shown for a variety of mouse tissues and organs. Complete results of GO analysis are in Table S6. FDR (Benjamini and Hochberg 1995) corrected –log10p-values are plotted.

We compared MAE genes inferred using MaGIC in mouse CD43^−^ B cells and human peripheral blood mononuclear cells, both of the lymphoid lineage (Figure 2A). There were 7429 unambiguous orthologous gene pairs between the two genomes, for which MAE or BAE could be inferred in both mouse and human cells (Table S5). Of these, 563 mouse genes (p_mouse_ = 563/7429 = 0.076) and 580 human genes (p_human_ = 0.078) were classified as MAE. If the identities of MAE genes were completely independent between mouse and human, we would expect 44 of these genes (p_mouse_ × p_human_ × 7429) to be MAE in both species. Instead, we observed 240 orthologous gene pairs in which MAE was conserved (Figure 2A; hypergeometric *P* = 3.9E-131). This finding suggests that the propensity of genes for MAE is largely conserved between human and mouse.

Using chromatin signature, we then inferred MAE in multiple mouse tissues for which ChIP-Seq and gene expression datasets were available, and compared over- and underrepresented GO categories to those previously reported (Nag *et al.* 2013) for MAE genes in human lymphoblasts (Figure 2B and Table S6). As in human, terms associated with embryonic development and cell surface proteins were preferentially enriched in MAE genes, whereas terms associated with housekeeping functions were depleted (Figure 2B).

Extensive commonalities between MAE in the human and mouse genomes imply that molecular mechanisms underlying this mode of gene regulation and possibly its functional impact(s) have been generally conserved since the last common ancestor of primates and rodents, 70−80 million years ago (Foote *et al.* 1999). This conservation suggests that experiments using the mouse model will further our understanding the role of MAE in human.

### Chromatin signature profiles are consistent with lineage-specific establishment and stable maintenance of MAE

The process of MAE establishment is poorly understood. Although MAE maintenance can be extremely stable (Gimelbrant *et al.* 2005), it is not clear how cell differentiation affects the MAE state. Recent studies, using clonal embryonic stem cells from highly polymorphic mice, indicated that in a neuronal lineage, MAE is already established by the progenitor stage and, for a limited number of tested genes, maintained in neurons (Eckersley-Maslin *et al.* 2014; Gendrel *et al.* 2014). We hypothesized that chromatin signature analysis of multiple cell lineages would shed light on the genome-wide patterns of MAE in developmental transitions.

**Figure 3:**
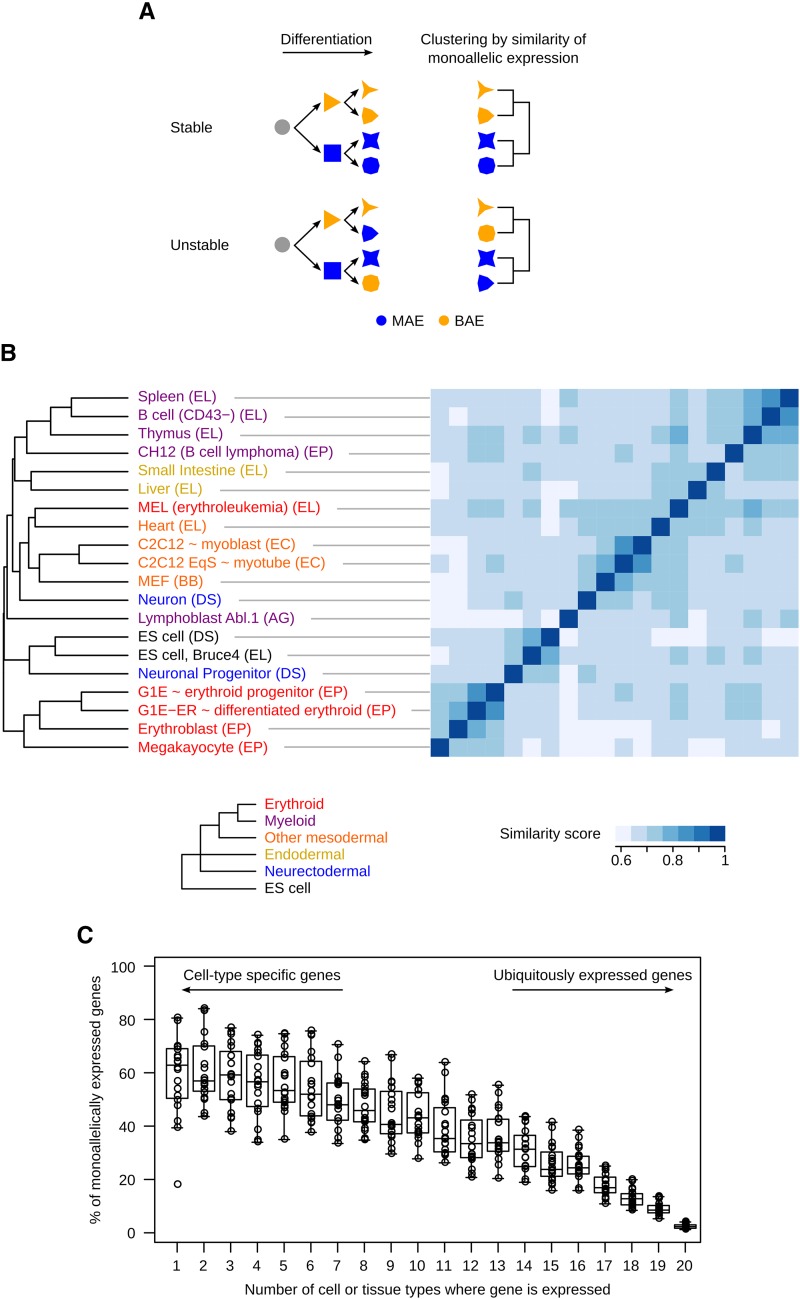
Comparison of mosaic monoallelic expression (MAE) chromatin signature profiles across tissues and cell types in mouse. (A) Schematic showing the hypothetical clustering of cell types (represented by different shapes) based on similar MAE state for a particular gene, in the case where MAE is stable throughout differentiation, and in the case where it is not. (B) Comparison of MAE profiles inferred from chromatin signature across analyzed tissues and cell types. Similarity scores were calculated for each pairwise comparison using only genes informative in both samples (similarity score scale at the bottom). Average-linkage clustering was performed using Gower’s distance (see the section *Materials and Methods*). Sample lineages are reflected in the color of the sample name, as follows: endodermic (yellow), ectodermic (cyan), lymphoid (purple), myeloid (red), other mesodermic (orange), and embryonic stem cells (black). The expected clustering of these lineages according to their embryonic origin is also shown (bottom). Laboratories that generated the ChIP-Seq datasets are indicated in parenthesis: ENCODE/LICR (EL), ENCODE/PSU (EP), Bernstein (BB), ENCODE/Caltech (EC), Schübeler (DS), and this work (AG). (C) Proportion of MAE genes inferred from chromatin signature among cell-type specific and ubiquitously expressed genes. Genes were grouped by the number of tissues where they are expressed. Then, in each bin, the proportion of MAE genes was calculated for each tissue and plotted individually (circles) and overall (Tukey boxplot).

We reasoned that if, once established in progenitor cells, MAE is stably maintained during differentiation, then cell types originating from the same progenitors should have more similar MAE profiles compared with each other than compared with cell types originating from different progenitors. Clustering based on the similarity of MAE profiles should, therefore, recapitulate embryonic lineage. Conversely, if genes that are MAE in progenitors frequently become BAE upon differentiation, then there should not be a strong relationship between MAE profile and embryonic lineage (Figure 3A).

Using chromatin signature, we mapped MAE profiles in 20 mouse organs and cell types, representing all three germ layers, obtained in most cases from inbred animals, and, with the exception of Abl.1 clone, consisting of nonclonal cell populations (Figure 3B, Table S1, and Table S2). Hierarchical clustering of MAE profiles showed that cell or tissue types of similar embryonic origin are more similar to each other than to those from distinct lineage. This is the case for lymphoid (CD43^‒^ B cells, CH12 cells, spleen, thymus), myeloid (megakaryocytes, erythroblasts, G1E and G1E-ER cells), other mesodermic (MEF, heart, and C2C12 cells) and endodermic (liver, small intestine) cells. Interestingly, neuronal progenitor cells were more similar to the embryonic stem cells from which they were differentiated than to the neurons derived from them.

Notably, samples from related tissues processed by different laboratories clustered together, suggesting that clustering reflects biological similarities rather than potential technical variation. Biological replicates for the same cells or tissue are also more similar to each other than to distinct cell or tissue types, indicating that differences in MAE related to lineage are more important than those caused by inter-individual or technical variability (Figure S4 and Table S7). Overall, our observations support lineage-specific establishment of MAE and its stable maintenance in differentiated tissues, while the precise timing of MAE establishment may vary depending on genes and cell types.

Consistent with lineage specificity of MAE, we also observed that a majority of cell-type specific genes display a MAE chromatin signature, but very few ubiquitously expressed genes do so (Figure 3C and Figure S5A). This trend also is observed after applying a correction for the imperfect accuracy of the MaGIC pipeline (Figure S5B) and is in agreement with the GO terms associated with MAE (Figure 2B), notably the underrepresentation of housekeeping activities. Altogether, our observations imply that MAE is linked to cell identity. This leads us to speculate that MAE plays an active role in determining cell identity, extending the principle behind two well-studied examples: allelic exclusion in immunoglobulin loci (Pernis *et al.* 1965) and monoallelic expression of olfactory receptor genes (Chess *et al.* 1994).

## Discussion

Using a combination of experimental and computational approaches, we showed that the presence of the H3K27me3/H3K36me3 gene–body chromatin signature is an informative proxy of MAE in mouse cells of lymphoid, mesenchymal, and neuroectodermal lineages. Combined with our studies in human cells, our findings suggest that the chromatin signature is an informative proxy of MAE in many, perhaps all, mammalian somatic cell types.

The accuracy of the MaGIC approach makes it suitable to identify candidate MAE genes for functional or mechanistic studies, as well as to investigate genome-wide trends associated with MAE genes. The analyses made in this article thus provide a valuable resource for future studies on monoallelic expression. It should be noted, however, that a significant fraction of MAE genes is not identified by the H3K27me3/H3K36me3 chromatin signature. We, therefore, expect that a more inclusive chromatin signature may be revealed by additional datasets for distinct chromatin marks, which might delineate subgroups of MAE genes with distinct properties. In support of this possibility, among MAE genes identified by RNA-Seq, genes with the H3K27me3/H3K36me3 chromatin signature (true positives) are enriched in GO terms related to cell-membrane proteins compared to MAE genes without this signature (false negatives); furthermore, genes in the true positive group tend to be expressed at a lower level compared with genes in the false negative group (not shown).

In addition, genes that are highly expressed in a subset of cells but repressed in others may display, at the cell population level, a chromatin signature similar to that of MAE genes. This is also the case of genes with dynamic expression, if changes in expression are reflected by variations in gene-body H3K27me3 or H3K36me3 enrichment. Such patterns of expression could partly explain differences between chromatin signature and allelic expression measurements, in addition to the technical considerations listed in the *Results* section. Although concerning a minority of genes, this possible confounding effect should be taken into consideration when interpreting MaGIC results. Orthogonal approaches, such as RNA-fluorescence in situ hybridization or knock-in reporters, should be used for confirmation of MAE status of specific genes of interest.

That the MAE status of a mouse gene is predictive of its human ortholog’s MAE status (and vice versa) strongly suggests that the propensity for MAE is encoded in the DNA sequence—no other transmission mechanism is likely to persist over the evolutionary span separating rodents and primates. It further suggests that MAE is either conserved for its own functional effect, or is a consequence of another conserved property of these genes.

The identification of genetic elements controlling MAE could take advantage of the fact that the MAE chromatin signature is informative in mouse cells of inbred genetic background. For instance, chromatin signature could be used to identify genes that are MAE in one particular mouse strain but not another, which could be linked to sequence differences within regulatory elements between the two strains. These elements could then be tested by targeted mutagenesis, opening the way to precise investigation of MAE mechanism and role.

Our analyses showed that genes subject to MAE tend to have tissue-specific expression and encode cell surface proteins, including diverse signaling molecules. We thus expect that MAE primarily affects processes at the interface between cells and their environment, which includes neighboring cells, signaling molecules, and infectious agents. Accordingly, we further propose that an important biological consequence of MAE is generation of substantial functional mosaicism involving otherwise similar cells within mammalian tissues.

## 

## Supplementary Material

Supporting Information
