# Supplementary material for: Chromatin Signature Identifies Monoallelic Gene Expression Across Mammalian Cell Types
Source: G3 (Bethesda). 2015 Jun 18;5(8):1713–20. doi: 10.1534/g3.115.018853 (PMC4528328; doi:10.1534/g3.115.018853)
Supplement: Supporting Information [file supp_5_8_1713__index.html]

Chromatin Signature Identifies Monoallelic Gene Expression Across Mammalian Cell Types — Supporting Information 

# Chromatin Signature Identifies Monoallelic Gene Expression Across Mammalian Cell Types

## Supporting Information for Nag *et al.*, 2015

**Files in this Data Supplement:**

- Supporting Information - Figures S1-S5 and descriptions of Tables S1-S7 (PDF, 2 MB)
- Figure S1 - Distribution of genes in the chromatin signature space and MAE classifier. (PDF, 1 MB)
- Figure S2 - Effect of gene length and expression level filtering on chromatin based MAE inference using MaGIC pipeline. (PDF, 456 KB)
- Figure S3 - Example dataset excluded from analysis due to low ChIP-Seq dynamic range. (PDF, 438 KB)
- Figure S4 - Comparison of MAE profiles between biological replicates. (PDF, 580 KB)
- Figure S5 - Cell-type specificity of MAE patterns in mouse. (PDF, 504 KB)
- Table S1 - Data sources. Information is provided for each dataset used in this study, organized by tissue or cell-type (first tab) and by dataset (second tab). (.zip, 23 KB)
- Table S2 - Mouse chromatin signature and MaGIC predictions. (.zip, 11 MB)
- Table S3 - Allelic expression. (.zip, 2 MB)
- Table S4 - Human chromatin signature and MaGIC predictions. (.zip, 1 MB)
- Table S5 - Analysis of orthologous MAE genes in human and mouse. Description of the table is provided in the first sheet. (.zip, 249 KB)
- Table S6 - GO analysis of the MAE genes across tissues. Description of the table is provided in the first sheet. (.zip, 96 KB)
- Table S7 - Mouse chromatin signature and MaGIC predictions for biological replicates. (.zip, 17 MB)
