# Supplementary material for: Chromatin Signature Identifies Monoallelic Gene Expression Across Mammalian Cell Types
Source: G3 (Bethesda). 2015 Jun 18;5(8):1713–20. doi: 10.1534/g3.115.018853 (PMC4528328; doi:10.1534/g3.115.018853)
Supplement: Supporting Information [file supp_g3.115.018853_018853SI.pdf]

G3: Genes | Genomes | Genetics

Chromatin signature identifies monoallelic gene expression across mammalian cell types

Anwesha Nag*[1], Sébastien Vigneau*[1], Virginia Savova*, Lillian M. Zwemer*, Alexander A. Gimelbrant*[2]

* Department of Cancer Biology and Center for Cancer Systems Biology, Dana-Farber Cancer Institute; Department of Genetics, Harvard Medical School; Boston, MA 02115

[1] Equal contribution

[2] Corresponding author: Alexander A. Gimelbrant, Dana-Farber Cancer Institute, Smith SM922B, 450 Brookline Ave, Boston, MA 02115. E-mail: gimelbrant@mail.dfci.harvard.edu

DOI: 10.1534/g3.115.018853

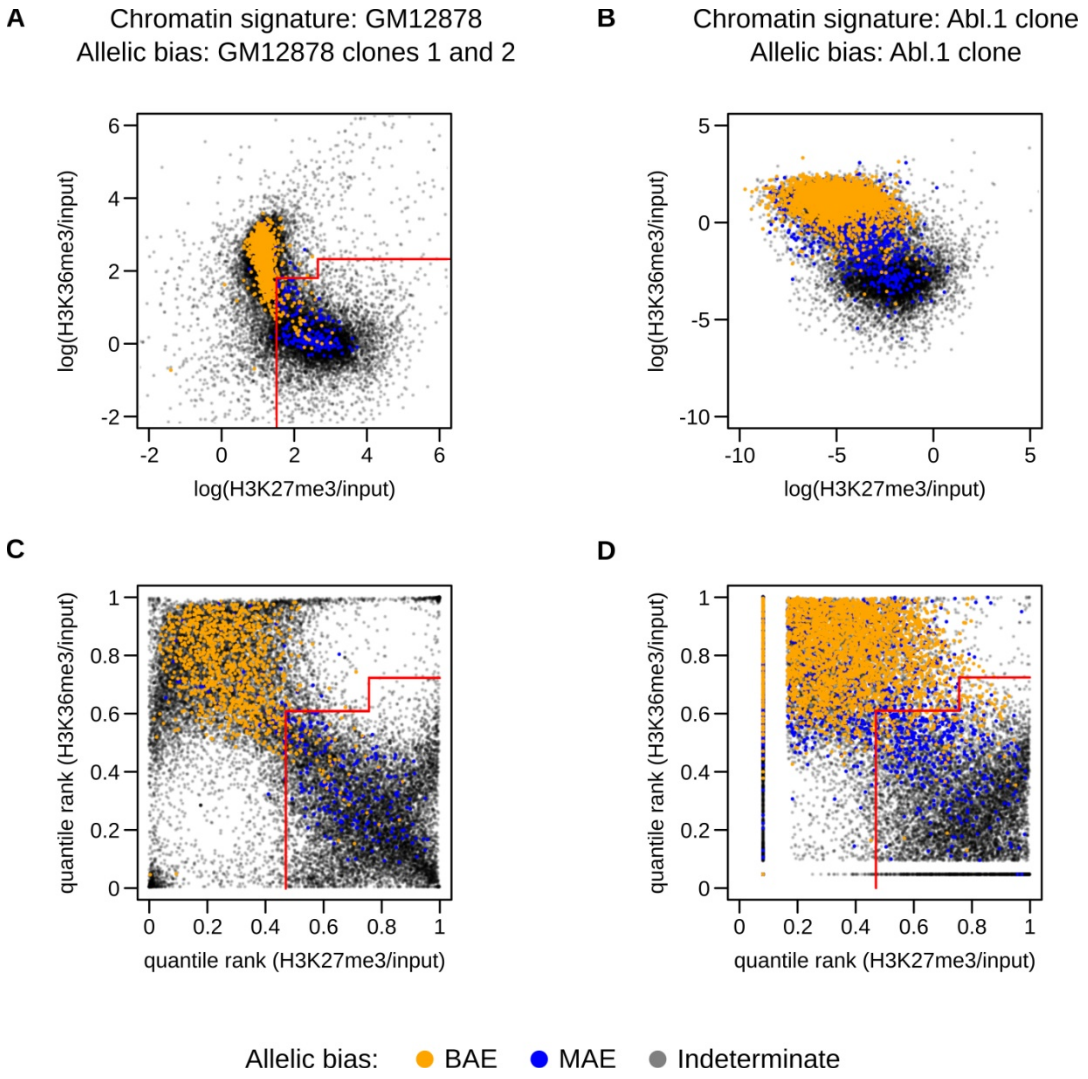

Figure S1 Distribution of genes in the chromatin signature space and MAE classifier. (A, B) Genes are plotted according to the log2 of H3K27me3 and H3K36me3 signals in their gene body, normalized to input. (C, D) Genes are plotted according to the quantile rank of H3K27me3 and H3K36me3 signals normalized to input. (A-D) Genes are plotted in orange, blue or grey if they are called as BAE, MAE or undetermined, respectively, based on allelic expression bias (Table S3). When meaningful, the MAE classifier delineation is drawn in red. Distribution of genes in the H3K27me3/H3K36me3 space is compared between human GM12878 dataset, used to train the classifier (A, C; Table S4), and mouse Abelson lymphoblast clone 1 dataset (B, D; Table S2). Both datasets show a two-lobes distribution when plotted in the log2 space (A, B), with the bottom-right lobe corresponding predominantly to MAE genes. The scales are, however, drastically different, precluding the use of the human-trained classifier with the mouse dataset in that space. The same data plotted in the quantile rank space (C, D) reproduce the two-lobes distribution as higher gene density in the top-left and bottom-right corners, the latter being enriched in MAE genes. In this case, the same classifier can be used with both datasets because the scales are identical. Note that genes with no detectable ChIP-Seq enrichment are given an average rank that appears as a horizontal or vertical alignment in the plot (D).

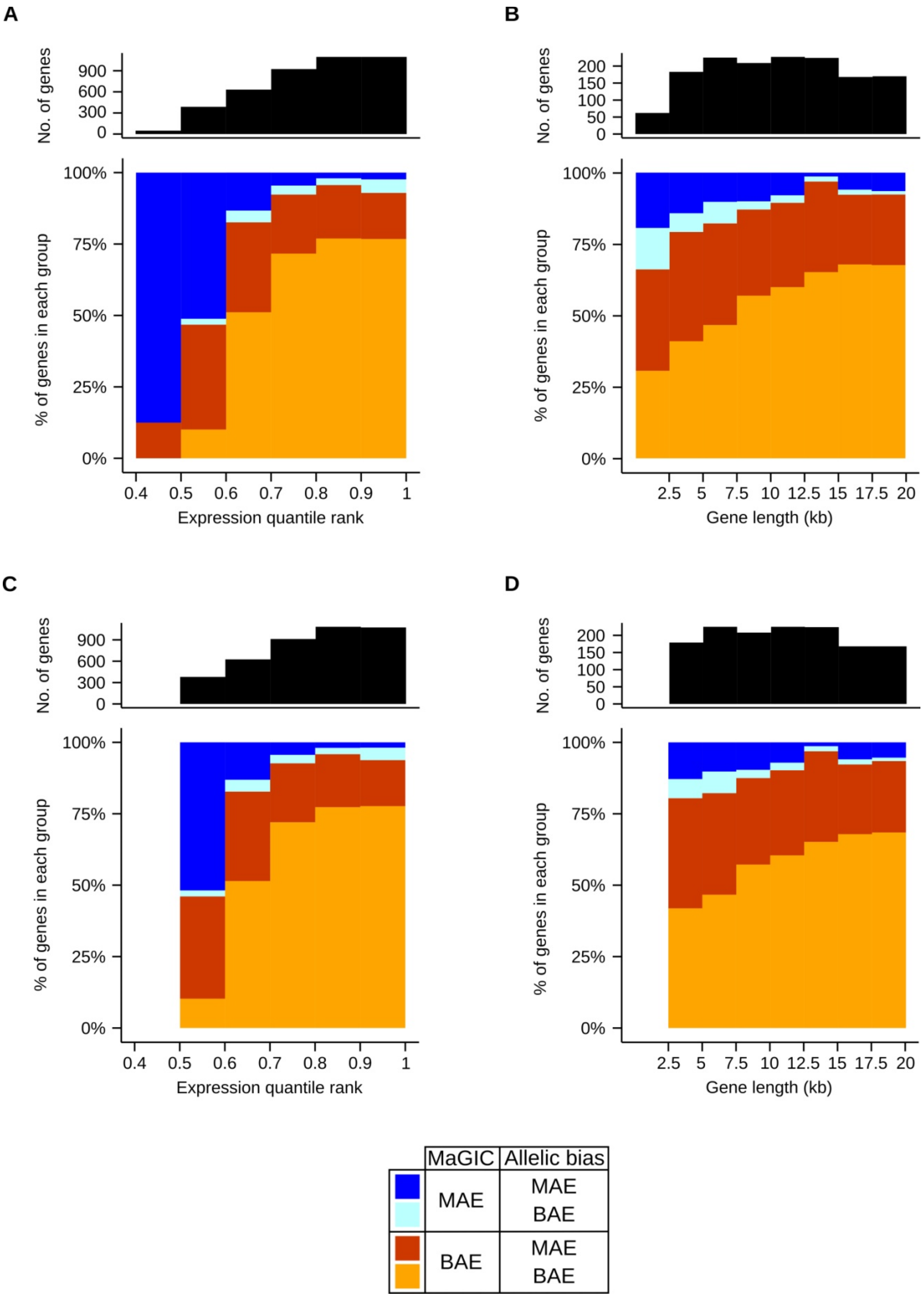
A
B
C
D
No. of genes
% of genes in each group
0%
25%
50%
75%
100%
0.4
0.5
0.6
0.7
0.8
0.9
1
Expression quantile rank
2.5
5
7.5
10
12.5
15
17.5
20
Gene length (kb)
MaGIC
Allelic bias
MAE
BAE

Figure S2 Effect of gene length and expression level filtering on chromatin based MAE inference using MaGIC pipeline. ChIP-Seq, RNA abundance and allelic expression bias data are from Abl.1 clone (Tables S2, S3). (A, B) MAE status was inferred using only normalized ChIP-Seq signal, with no additional filter. (C, D) Filters for both expression level (greater than median expression) and gene length (>2.5 kb) were applied to the data in A and B. (A-D, bottom panels) Percentage of genes with consistent or inconsistent MAE/BAE classification using MaGIC and allelic bias (color code indicated on figure), plotted according to their expression rank (A, C) and gene length (B, D). (A-D, top panels) Number of genes in each bin. Note: No genes with expression rank lower than 0.4 had allelic bias calls.

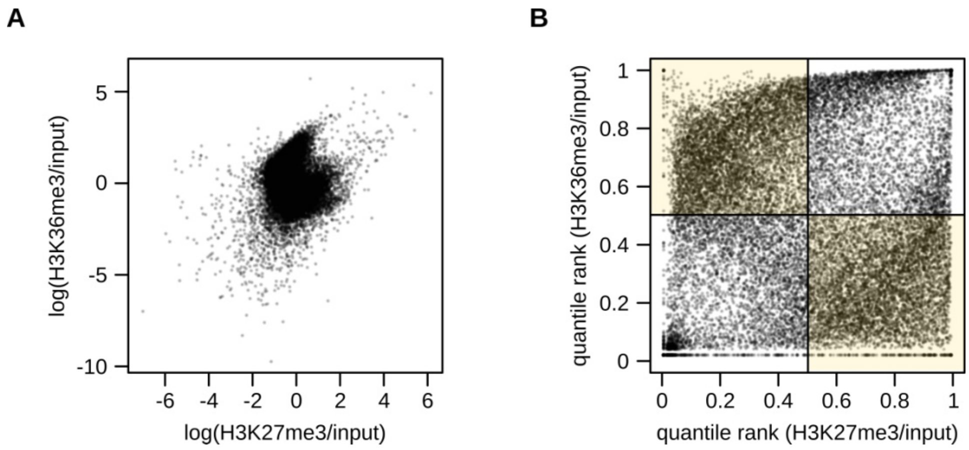

Figure S3 Example dataset excluded from analysis due to low ChIP-Seq dynamic range. Genes are plotted according to the log2 (A) or quantile rank (B) of ChIP-Seq H3K27me3 and H3K36me3 signals in their gene body normalized to input, in adult mouse kidney (GSE31039; Table S1, S2). The distribution of genes does not show clearly distinct lobes in the log2 space (A) and only 59% of genes (less than the required 60%) are contained in the top-left and bottom-right median-bound quadrants (yellow shade) in the quantile rank space. Since this distribution deviates from those with which the classifier was tested (Figure 1), we excluded the dataset from further analysis.

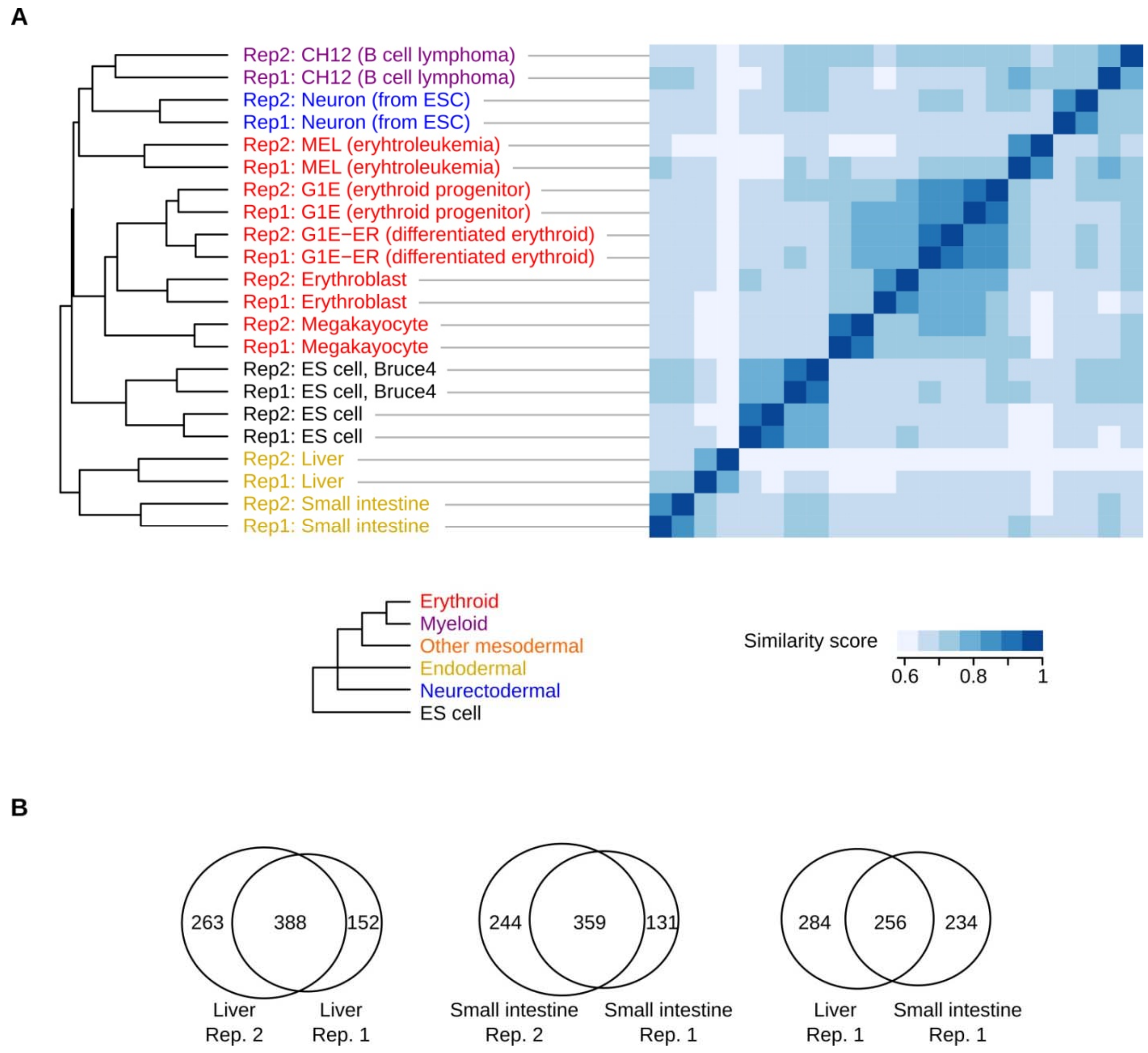

Figure S4 Comparison of MAE profiles between biological replicates. (A) Comparison of MAE profiles inferred from chromatin signature between cell or tissue types, and between biological replicates of the same cell or tissue type (Table S7). Clustering was performed as described in figure 3 and shows that MAE profiles are more similar between biological replicates than between even closely related cell or tissue types. (B) Example comparison of inferred MAE profiles between biological replicates of the same tissue, and between different tissues. Venn Diagrams indicate the number of MAE genes that are shared or unique between biological replicates from liver (left) and small intestine (center), or when comparing both organs (right) (Table S7).

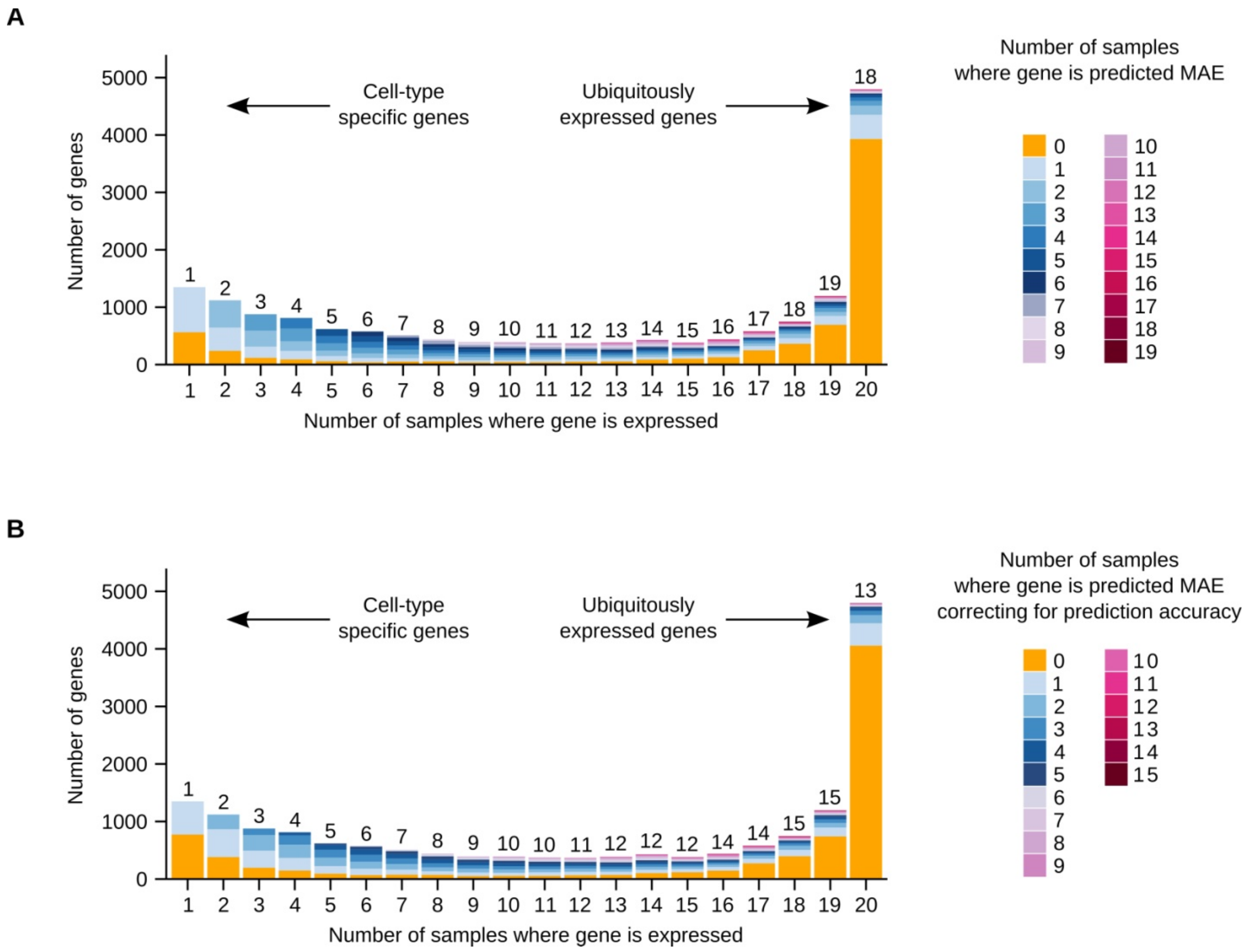

Figure S5 Cell-type specificity of MAE patterns in mouse. (A, B) Distribution of MAE and BAE genes among cell-type specific and ubiquitously expressed genes. Genes were considered “expressed” in a given sample if their expression value in that sample was above median for all autosomal genes. Genes expressed in only one tissue are in the leftmost bin; genes expressed in all assessed tissues are in the rightmost. Within each bin, color coded areas represent the number of genes that show MAE signature in the noted number of samples (see color code key to the right). Over each bin is shown the maximum number of samples with MAE of the same gene. Values are given without correction (A) or with correction for MaGIC accuracy as described in Materials and methods (B).

Tables S1-S7
Available for download at www.g3journal.org/lookup/suppl/doi:10.1534/g3.115.018853/-/DC1

Table S1 Data sources. Information is provided for each dataset used in this study, organized by tissue or cell-type (first tab) and by dataset (second tab).

Table S2 Mouse chromatin signature and MaGIC predictions. The table indicates for each tissue or cell-type and for each gene, the genomic coordinates in mm8 or mm9 assembly, the quantile rank of H3K27me3 and H3K36me3 normalized to input (column names ending with "rank_K27divIN" and "rank_K36divIN", respectively), the expression quantile rank (column names ending with "rank_expr"), the prediction based on chromatin signature (column names ending with "isMAE", with value of 1 if gene was called MAE and 0 if it was called BAE), and whether genes passed the filter for gene length greater than 2.5 kb and expression greater than median (column names ending with "passFilter", with value of 1 if filter was p assed, and 0 otherwise). Note that similar information for individual biological replicate is provided in **Table S7**. In addition, the "Imprinted" column indicates whether genes are imprinted in mouse (value of 1) or not (value of 0) according to GeneBook (http://www.mousebook.org/imprinting-gene-list) and geneimprint (http://www.geneimprint.com/site/genes-by-species.Mus+musculus) databases.

Table S3. Allelic expression. The table indicates for each clone included in the study and for each gene, the read count per SNP (column names ending with "SNP_count"), the result of the equivalence test (column names ending with "Equivalence", with value of 1 if equivalent, -1 if not equivalent, and 0 if undetermined), the allelic bias across all SNPs (column names ending with "Bias"), the binomial test p-value (column names ending with "Binom_p"), whether the gene is called MAE (column names ending with "isMAE", with value of 1 if called MAE and 0 otherwise), and whether the gene is called BAE (column names ending with "isBAE", with value of 1 if called BAE and 0 otherwise). In addition, the columns "Abl_isMAE", "Fib_isMAE", and "NP_Gendrel_isMAE" indicate whether the gene is MAE in at least one clone of the corresponding cell type (value of 1 if MAE and 0 otherwise). The columns "Abl_isBAE", "Fib_isBAE", and "NP_Gendrel_isBAE", indicate whether the gene is BAE in at least one clone and MAE in no clone of the corresponding cell type (value of 1 if BAE and 0 otherwise). "Abl" refers to lymphoblast clones, "Fib" refers to fibroblast clones, and "NP_Gendrel" refers to neuronal progenitor clones from (Gendrel et al. 2014), as detailed in **Table S1**.

Table S4. Human chromatin signature and MaGIC predictions. The table indicates for each cell-type and for each gene, the genomic coordinates in hg19 assembly, the quantile rank of H3K27me3 and H3K36me3 normalized to input (column names ending with "rank_K27divIN" and "rank_K36divIN", respectively), the prediction based on chromatin signature (column names ending with "isMAE", with value of 1 if gene was called MAE and 0 if it was called BAE) as well as, when used in the article, the expression quantile rank (column names ending with "rank_expr") and whether genes passed the filter for gene length greater than 2.5 kb and expression greater than median (column names ending with "passFilter", with value of 1 if filter was passed, and 0 otherwise).

Table S5. Analysis of orthologous MAE genes in human and mouse. Description of the table is provided in the first sheet.

Table S6. GO analysis of the MAE genes across tissues. Description of the table is provided in the first sheet.

Table S7. Mouse chromatin signature and MaGIC predictions for biological replicates. The table indicates for each tissue or cell-type and for each gene, the genomic coordinates in mm8 and mm9 assembly, the quantile rank of H3K27me3 and H3K36me3 normalized to input (column names ending with "rank_K27divIN" and "rank_K36divIN", respectively), the expression quantile rank (column names ending with "rank_expr"), the prediction based on chromatin signature (column names ending with "isMAE", with value of 1 if gene was called MAE and 0 if it was called BAE), and whether genes passed the filter for gene length greater than 2.5 kb and expression greater than median (column names ending with "passFilter", with value of 1 if filter was passed, and 0 otherwise).
