# Supplementary material for: Chromatin Signature Identifies Monoallelic Gene Expression Across Mammalian Cell Types
Source: G3 (Bethesda). 2015 Jun 18;5(8):1713–20. doi: 10.1534/g3.115.018853 (PMC4528328; doi:10.1534/g3.115.018853)
Supplement: Supporting Information [file supp_g3.115.018853_FigureS1.pdf]

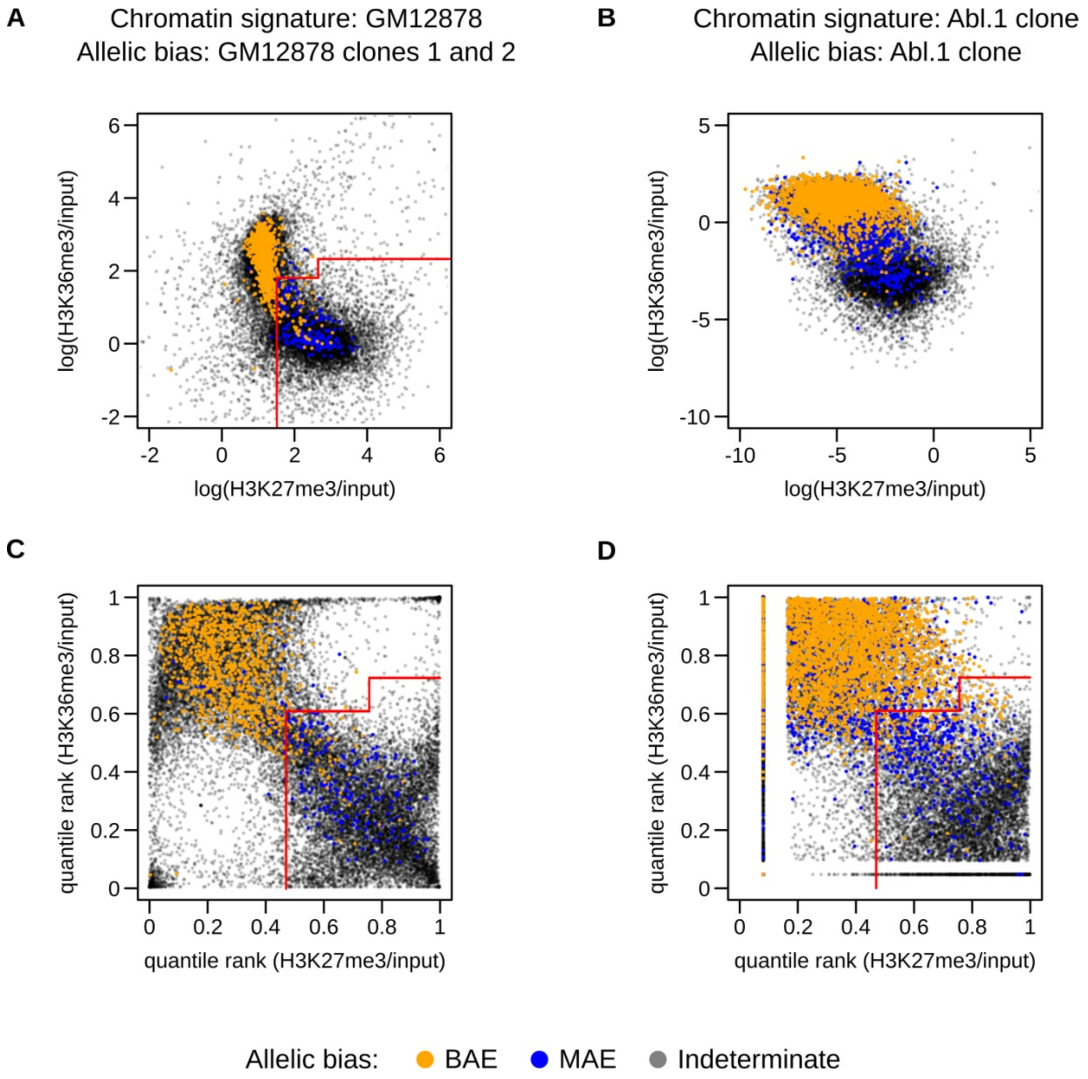

Figure S1 Distribution of genes in the chromatin signature space and MAE classifier. (A, B) Genes are plotted according to the log2 of H3K27me3 and H3K36me3 signals in their gene body, normalized to input. (C, D) Genes are plotted according to the quantile rank of H3K27me3 and H3K36me3 signals normalized to input. (A-D) Genes are plotted in orange, blue or grey if they are called as BAE, MAE or undetermined, respectively, based on allelic expression bias (Table S3). When meaningful, the MAE classifier delineation is drawn in red. Distribution of genes in the H3K27me3/H3K36me3 space is compared between human GM12878 dataset, used to train the classifier (A, C; Table S4), and mouse Abelson lymphoblast clone 1 dataset (B, D; Table S2). Both datasets show a two-lobes distribution when plotted in the log2 space (A, B), with the bottom-right lobe corresponding predominantly to MAE genes. The scales are, however, drastically different, precluding the use of the human-trained classifier with the mouse dataset in that space. The same data plotted in the quantile rank space (C, D) reproduce the two-lobes distribution as higher gene density in the top-left and bottom-right corners, the latter being enriched in MAE genes. In this case, the same classifier can be used with both datasets because the scales are identical. Note that genes with no detectable ChIP-Seq enrichment are given an average rank that appears as a horizontal or vertical alignment in the plot (D).
