# Supplementary material for: Chromatin Signature Identifies Monoallelic Gene Expression Across Mammalian Cell Types
Source: G3 (Bethesda). 2015 Jun 18;5(8):1713–20. doi: 10.1534/g3.115.018853 (PMC4528328; doi:10.1534/g3.115.018853)
Supplement: Supporting Information [file supp_g3.115.018853_FigureS2.pdf]

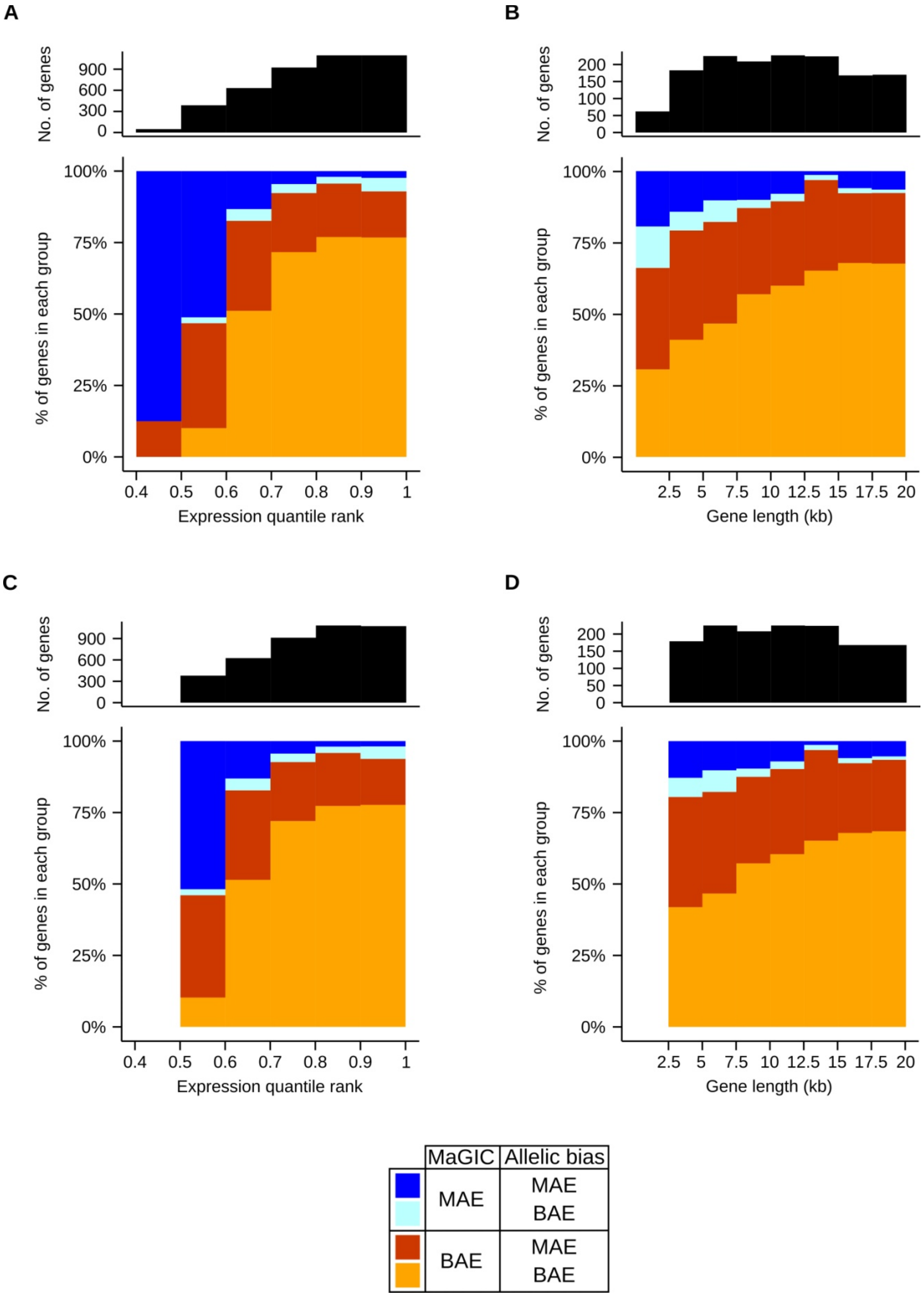
A
No. of genes
% of genes in each group
Expression quantile rank
B
No. of genes
% of genes in each group
Gene length (kb)
C
No. of genes
% of genes in each group
Expression quantile rank
D
No. of genes
% of genes in each group
Gene length (kb)
MaGIC
Allelic bias
MAE
MAE
BAE
BAE
MAE
BAE

Figure S2 Effect of gene length and expression level filtering on chromatin based MAE inference using MaGIC pipeline. ChIP-Seq, RNA abundance and allelic expression bias data are from Abl.1 clone (Tables S2, S3). (A, B) MAE status was inferred using only normalized ChIP-Seq signal, with no additional filter. (C, D) Filters for both expression level (greater than median expression) and gene length (>2.5 kb) were applied to the data in A and B. (A-D, bottom panels) Percentage of genes with consistent or inconsistent MAE/BAE classification using MaGIC and allelic bias (color code indicated on figure), plotted according to their expression rank (A, C) and gene length (B, D). (A-D, top panels) Number of genes in each bin. Note: No genes with expression rank lower than 0.4 had allelic bias calls.
