# Supplementary material for: Chromatin Signature Identifies Monoallelic Gene Expression Across Mammalian Cell Types
Source: G3 (Bethesda). 2015 Jun 18;5(8):1713–20. doi: 10.1534/g3.115.018853 (PMC4528328; doi:10.1534/g3.115.018853)
Supplement: Supporting Information [file supp_g3.115.018853_FigureS3.pdf]

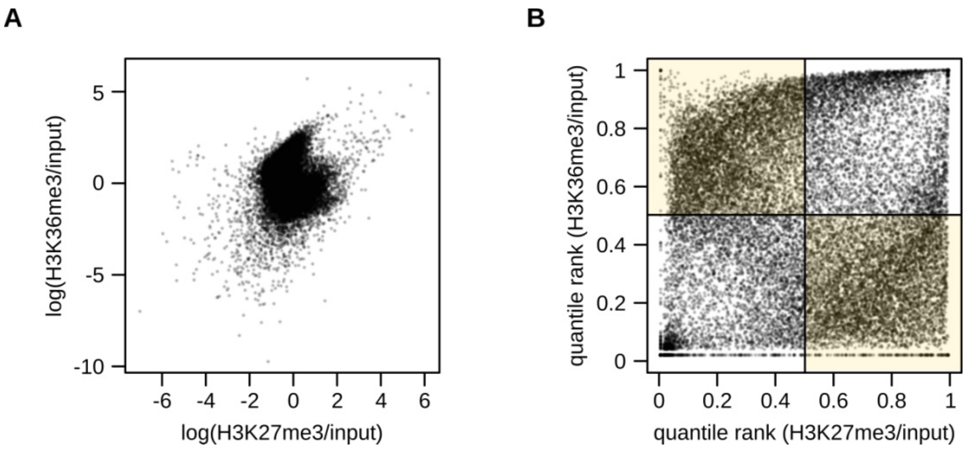

Figure S3 Example dataset excluded from analysis due to low ChIP-Seq dynamic range. Genes are plotted according to the log2 (A) or quantile rank (B) of ChIP-Seq H3K27me3 and H3K36me3 signals in their gene body normalized to input, in adult mouse kidney (GSE31039; Table S1, S2). The distribution of genes does not show clearly distinct lobes in the log2 space (A) and only 59% of genes (less than the required 60%) are contained in the top-left and bottom-right median-bound quadrants (yellow shade) in the quantile rank space. Since this distribution deviates from those with which the classifier was tested (Figure 1), we excluded the dataset from further analysis.
