# Supplementary material for: Chromatin Signature Identifies Monoallelic Gene Expression Across Mammalian Cell Types
Source: G3 (Bethesda). 2015 Jun 18;5(8):1713–20. doi: 10.1534/g3.115.018853 (PMC4528328; doi:10.1534/g3.115.018853)
Supplement: Supporting Information [file supp_g3.115.018853_FigureS4.pdf]

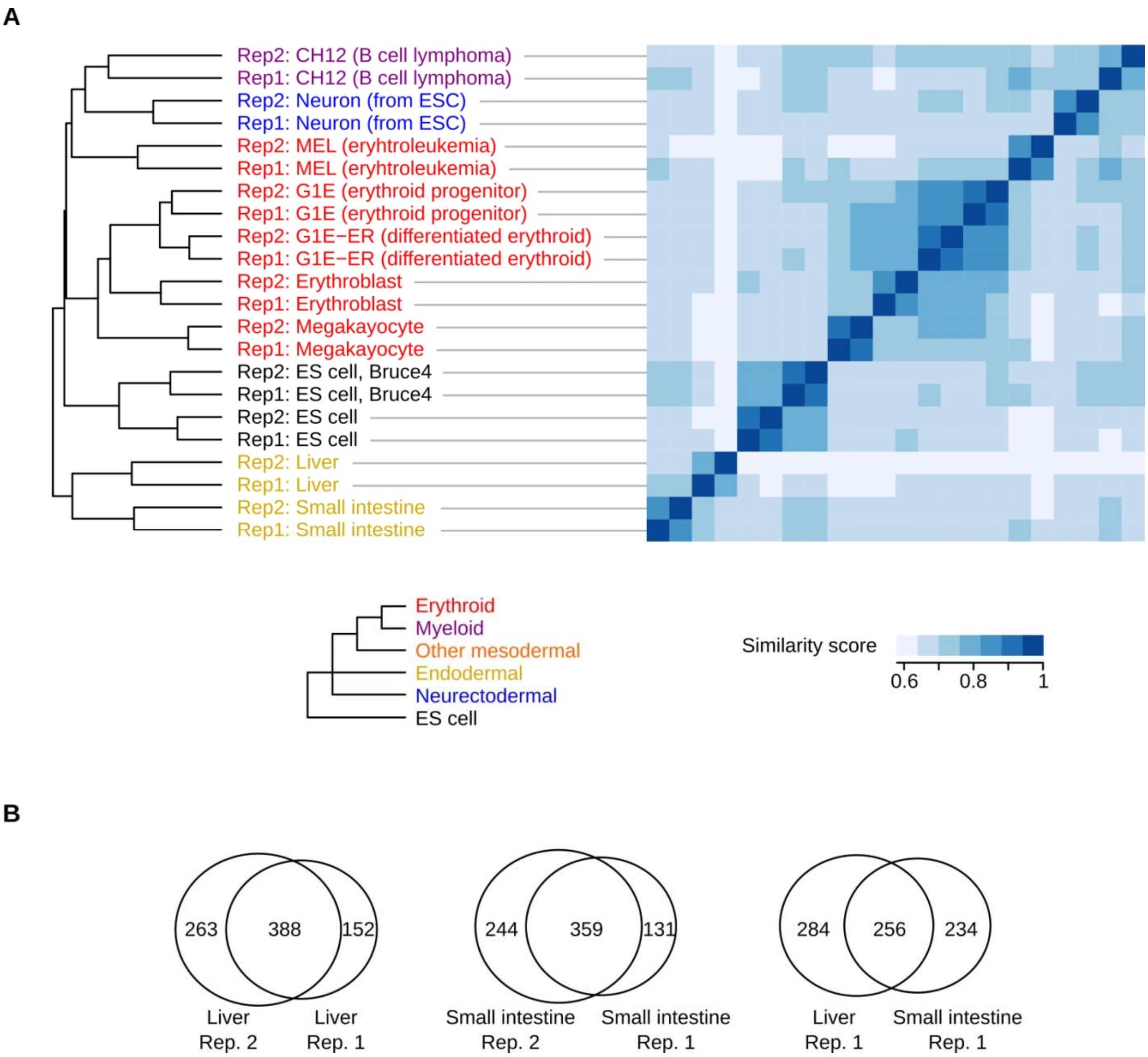

Figure S4 Comparison of MAE profiles between biological replicates. (A) Comparison of MAE profiles inferred from chromatin signature between cell or tissue types, and between biological replicates of the same cell or tissue type (Table S7). Clustering was performed as described in figure 3 and shows that MAE profiles are more similar between biological replicates than between even closely related cell or tissue types. (B) Example comparison of inferred MAE profiles between biological replicates of the same tissue, and between different tissues. Venn Diagrams indicate the number of MAE genes that are shared or unique between biological replicates from liver (left) and small intestine (center), or when comparing both organs (right) (Table S7).
