# Supplementary material for: Chromatin Signature Identifies Monoallelic Gene Expression Across Mammalian Cell Types
Source: G3 (Bethesda). 2015 Jun 18;5(8):1713–20. doi: 10.1534/g3.115.018853 (PMC4528328; doi:10.1534/g3.115.018853)
Supplement: Supporting Information [file supp_g3.115.018853_FigureS5.pdf]

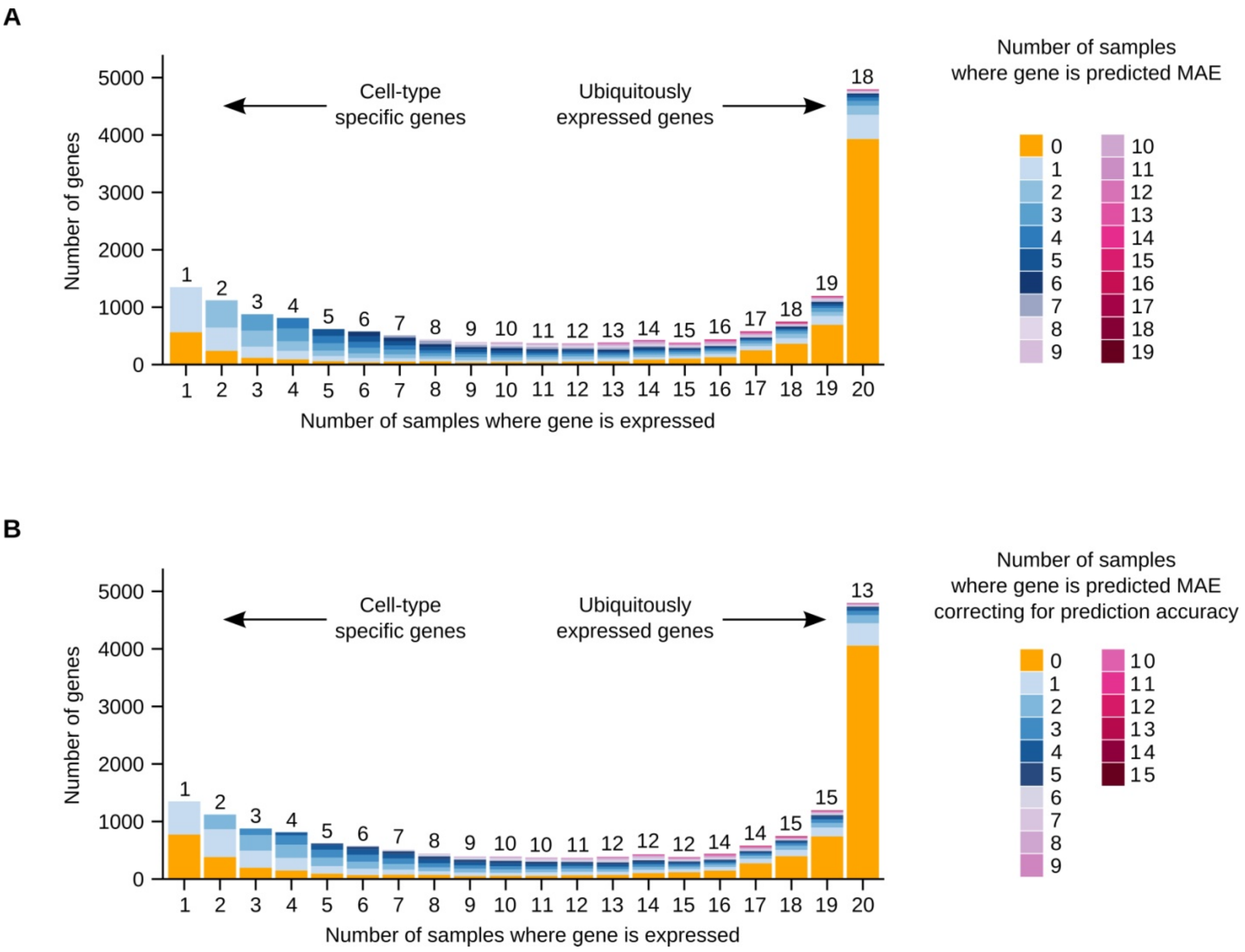

Figure S5 Cell-type specificity of MAE patterns in mouse. (A, B) Distribution of MAE and BAE genes among cell-type specific and ubiquitously expressed genes. Genes were considered “expressed” in a given sample if their expression value in that sample was above median for all autosomal genes. Genes expressed in only one tissue are in the leftmost bin; genes expressed in all assessed tissues are in the rightmost. Within each bin, color coded areas represent the number of genes that show MAE signature in the noted number of samples (see color code key to the right). Over each bin is shown the maximum number of samples with MAE of the same gene. Values are given without correction (A) or with correction for MaGIC accuracy as described in Materials and methods (B).
